# Phylogenetic analysis of the 5ʹ untranslated region of HCV from cirrhotic patients in Khyber Pakhtunkhwa, Pakistan

**DOI:** 10.1038/s41598-021-94063-1

**Published:** 2021-07-22

**Authors:** Amin Ullah, Irshad Ur Rehman, Jamshaid Ahmad, Margaret Odenthal, Saad Ahmad, Tariq Nadeem, Qurban Ali, Muhammad Rizwan, Muhammad Ajmal Khan, Said Hassan, Hina Ahsan, Bashir Ahmad

**Affiliations:** 1 Department of Microbiology and Biotechnology, Abasyn University Peshawar, Khyber Pakhtunkhwa, Pakistan; 2 Institute of Pathology, Laboratory of Translational Molecular Pathology, University Hospital of Cologne, Cologne, Germany; 3 Kabir Medical College Peshawar, Gandhara University Peshawar, Khyber Pakhtunkhwa, Pakistan; 4 Centre of Excellence in Molecular Biology, University of the Punjab Lahore, Lahore, Pakistan; 5 Institute of Molecular Biology and Biotechnology, The University of Lahore, Lahore, Pakistan; 6 Department of Biotechnology and Microbiology, Swat University, Khyber Pakhtunkhwa, Pakistan; 7 Division of Life Sciences, Center for Cancer Research and State Key Lab for Molecular Neuroscience, Hong Kong University of Science and Technology, Clear Water Bay, Hong Kong; 8 Institute of Biotechnology and Microbiology, Bacha Khan University Charsadda, Khyber Pakhtunkhwa, Pakistan; 9 Riphah Faculty of Pharmaceutical Sciences, G7/4 7th Avenue, Islamabad, Pakistan

**Keywords:** Diseases, Infectious diseases

## Abstract

Hepatitis C virus (HCV), a small, single-stranded RNA virus with a 9.6 kb genome, is one of the most common causes of liver diseases. Sequencing of the 5ʹ untranslated region (UTR) is usually used for HCV genotyping, but it is less important in numerous subtypes due to its scarce sequence variations. This study aimed to identify genotypes using the 5ʹ UTR of HCV from cirrhotic patients of Khyber Pakhtunkhwa (KP). Serum RNA samples (44) were screened by real time PCR to determine the HCV viral load. Nested PCR was performed to identify cDNA and the 5ʹ UTR. The HCV 5′ UTR was sequenced using the Sanger method. MEGA-7 software was used to analyze evolutionary relatedness. After 5ʹ UTR sequencing, 26 samples (59%) were identified as genotype 3, and 2 samples (6%) were identified as genotypes 1, 2 and 4. The most predominant genotype was 3a, and genotype 4 was rarely reported in the phylogenetic tree. Analysis of the HCV 5ʹ UTR is an efficient alternative method for confirmation of various genotypes. Phylogenetic analysis showed that genotype 3 was dominant in the area of KP, Pakistan.

## Introduction

Liver diseases due to hepatitis C virus (HCV) pose serious health threats worldwide. HCV-induced hepatitis C has an alarming high frequency of progression to chronic liver disease (CLD), liver cirrhosis and carcinoma. Currently, more than 70 million people suffer from HCV-mediated CLD worldwide. Almost 400,000 people die due to cirrhosis and liver cancer, caused by HCV each year^1,2^. HCV is a lipoprotein-enveloped ribovirus with a 9.600 nucleotide 5ʹ–3ʹ UTR^3^. The RNA genome has an untranslated region (UTR), three structural (core, E1, E2) and seven nonstructural genes (p7, NS2-NS5). The nonstructural protein (NS5B) is a moderately variable region and is commonly used for HCV subtyping^4^. In terms of primary sequence and secondary structures, the 5′ and 3′ UTRs are the most conserved areas of HCV RNA.

The 5ʹ UTR contains reasonably variable areas inclusive of NS5A, which codes for a nonstructural protein^5^. The 5ʹ UTR consists of 341 nucleotides, and due to its 90% sequence identity, it is commonly used for genotype identification. The 5ʹ-UTR stem loop structure contains entry sites (IRES)^6^. Mutations do not usually occur in the 5-UTR, and sometimes compensatory mutations are developed to preserve the base-pairing shape and conserve the structural characteristics associated with translation efficiency. Recently, it has been found that the first 145 sequences of the 5ʹ-UTR play a significant role in the replication of HCV RNA^7^. NS5A also has inadequate natural amino acid variability, which conserves its useful characteristics in vivo^8^.

Other articles on sequencing substantiate the 5ʹ UTR (324–341 nucleotides long) as the least mutated region in the HCV genome and describe it remaining conserved in all HCV genotypes^9–11^. This high-grade conservation makes the 5ʹ UTR the region of choice for performing (RT)-PCR detection tests, such as the HCV amplicor test^12^. A number of genotyping schemes have been established and utilized in this region to obtain phylogenetic genotype information^13,14^. Sequencing data of the 5ʹ UTR and other regions, such as the NS-3, NS-4, core and NS-5, have been used in phylogenetic studies and genotyping of HCV^15–17^.

Geographically, HCV genotype 1 is prevalent in Europe, Japan and the USA^18^, whereas genotype 2 is found in Korea and Taiwan, and genotype 3 is detected in Pakistan, India and Thailand. Genotype 4 is the most frequent genotype in Saudi Arabia, Egypt, Syria, Iraq, Vietnam and Lebanon. Genotype 5 is found in South Africa, and genotype 6 is found in Vietnam^19–21^. The current study focused on the analysis of 5′ UTR sequencing and identification of genotypes by comparison with reference genotypes.

## Materials and methods

### RNA isolation and cDNA preparation

All samples were collected from HCV cirrhotic patients treated at the Hayatabad Medical Complex (HMC) and Khyber Teaching Hospital (KTH), Peshawar, during January 2017–May 2018. The current study was approved by the competent authorities of the institute, and the whole study was carried out according to the ethical guidelines given by the institute. RNA was isolated from serum by a QIAamp viral RNA kit in accordance with the manufacturer’s protocol and stored at − 80 °C.
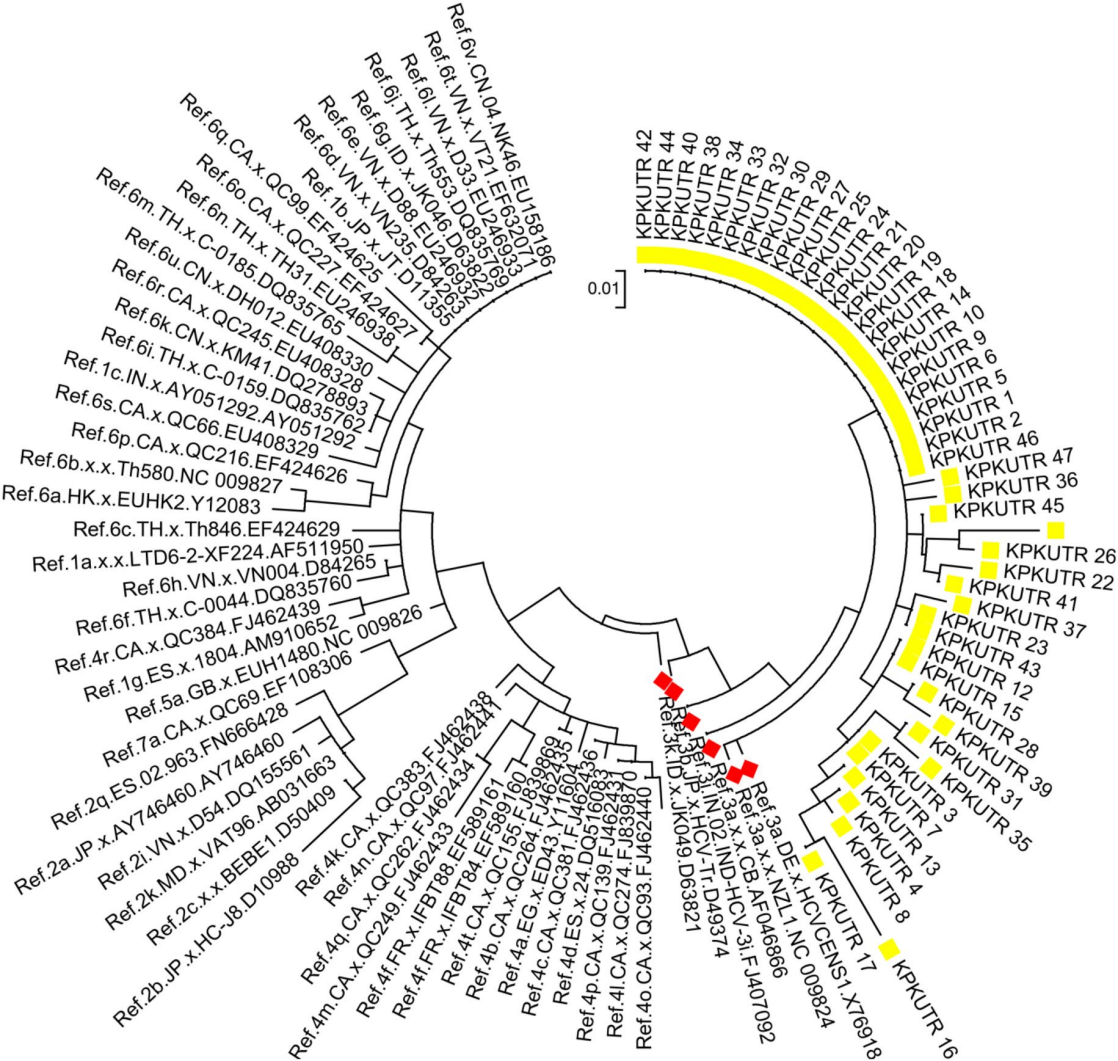


The cDNA was formed using 1 µl outer antisense primer (OAS), 10 µl RNA, 1 µl (200 U) M-MLV reverse transcriptase enzyme (BIORON Life Science cDNA Kit), 4 µl complete RT buffer, 2.5 µl PCR water, 1 µl dNTPs (10 mM) and 0.5 µl RNA inhibitors with a total volume of 20 µl. The following temperature cycle was then applied: 37 °C/60 min, 70 °C/10 s, and 22 °C/∞. The outer antisense primer was the reverse primer used in the first round of nested PCR amplification of the HCV 5ʹ UTR.

### PCR amplification

The 4 µl cDNA was amplified in the first round of nested PCR with forward and reverse primers of the 5′ UTR for HCV positivity. Similarly, 4 µl of the 1st round PCR product was amplified in the 2nd round PCR, with inner sense (IS) and inner anti sense (IAS) primers (Table 1). In both rounds of PCR, 7.1 µl PCR grade water, 6.9 µl master mix and 1 µl each primer were used with the following cycle: 94 °C/2 min initial denaturation and 35 cycles of 94 °C/30 s, 54 °C/30 s, and 72 °C/45 s for annealing with a final extension at 72 °C/10 min in a thermal cycler.

**Table 1:** HCV 5ʹ UTR primers and sizes.

Name	Primer	Base pairs
OS	5ʹctcttacgaggcgacactcc3ʹ	20
OAS	5ʹcaagcaccctatcaggcagt3ʹ	20
IS	5ʹgatcactcccctgtgaggaa3ʹ	20
IAS	5ʹctttcgcgacccaacactac3ʹ	20

### DNA sequencing

PCR products (5ʹ UTR) were purified and sequenced through a standard Sanger procedure on an ABI 3730XL DNA Sequencer at Macrogen sequencing services (South Korea). Chromas software (http://technelysium.com.au/wp/chromas/) was used to check and correct the sequence viewer. Nested PCR primers were used for sequencing (Table 1), and then sequences were submitted to BLAST to determine similarity to sample sequences in the reported databases. Sequences were submitted to GenBank, under the accession numbers MN038290-MN038122. Reference sequences of HCV genotypes were retrieved from the GenBank database to build a phylogenetic tree.

### Phylogenetic tree

Molecular Evolutionary Genetics Analysis software (MEGA version 7: http://www.megasoftware.net) was used to sequence the 5′ UTR, sequences were aligned by the maximum likelihood method and associated with the reference sequence of the identified genotype. The neighbor-joining algorithm of MEGA 7 was used to calculate the p-distance and the differences in each nucleotide sequence. The phylogenetic tree analysis utilized 1000 bootstrap resampling to test the robustness of the observed dominant clades.

### Statistical analysis

Statistical analysis was performed by IBM SPSS version 25, Windows 7 and Microsoft Excel version 13.

### Ethics approval

This study was approved by the Ethics Committee, Centre of Biotechnology and Microbiology, University of Peshawar, Pakistan. Written informed consent was obtained from all the individuals who participated in the study.

## Results

**Figure 1:**
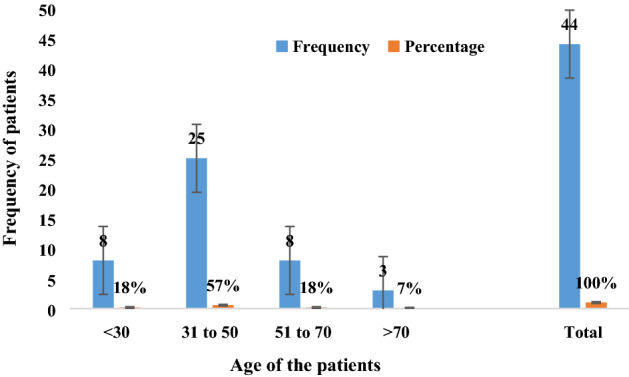
Frequency distribution of HCV cirrhotic patients based on age.

HCV cirrhotic samples (n = 44) were collected on the basis of their demographic variables. The average age and standard deviation (SD) of the patients was 48.69 ± 11.28 yrs. The numbers of male and female patients were n = 25 and 19, respectively. The highest viral load was recorded prior to and during the treatment > 200,0000 IU/mL, as shown in Table 2. A high number of HCV cirrhotic patients were identified or noted between the ages of 31–50 (Fig. 1).

**Table 2:** Baseline characteristics of HCV cirrhotic patients (n = 44).

Variable	No. of patients
**Gender**	
Male	25 (57%)
Female	19 (43%)
**Age**	
Mean ± SD	49.8 ± 12.33
Range	23–81
**Cirrhosis stage**	
Mild	27 (61%)
Moderate	10 (23%)
Gross	07 (16%)
**Treatment**	
Antiviral drugs	Sofusbuvir + ribavirin/daclatasvir
**ALT**	
Mean ± SD	105.87 ± 30.77
Range	87–283
**AFP**	
Mean ± SD	48.3 ± 39.80
Range	34–279
**Albumin**	
Mean ± SD	3.7 ± 0.4
**Bilirubin**	
Mean ± SD	1.4 ± 0.8
**AST**	
Mean ± SD	41.3 ± 29.60
Range	67- 209
**Viral load**	
< 200,0000 IU/mL	30
> 200,0000 IU/mL	14

*SD* standard deviation, *ALT* alanin transaminase, *AFP* alpha fetoprotein, *AST* asparagine transaminase.

**Figure 2:**
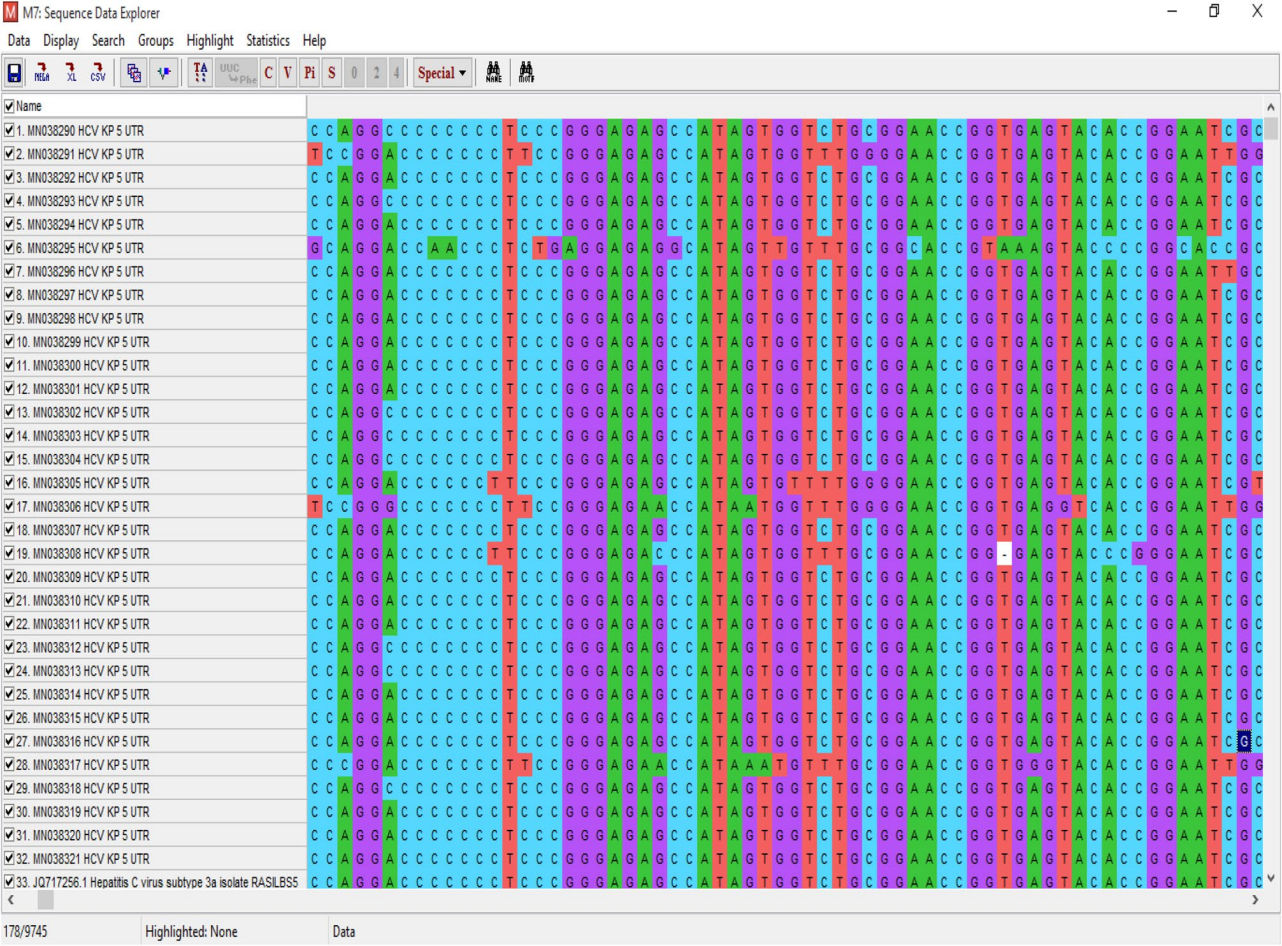
Alignment of the HCV 5ʹ UTR sequence with the reference sequence HCV.

**Figure 3:**
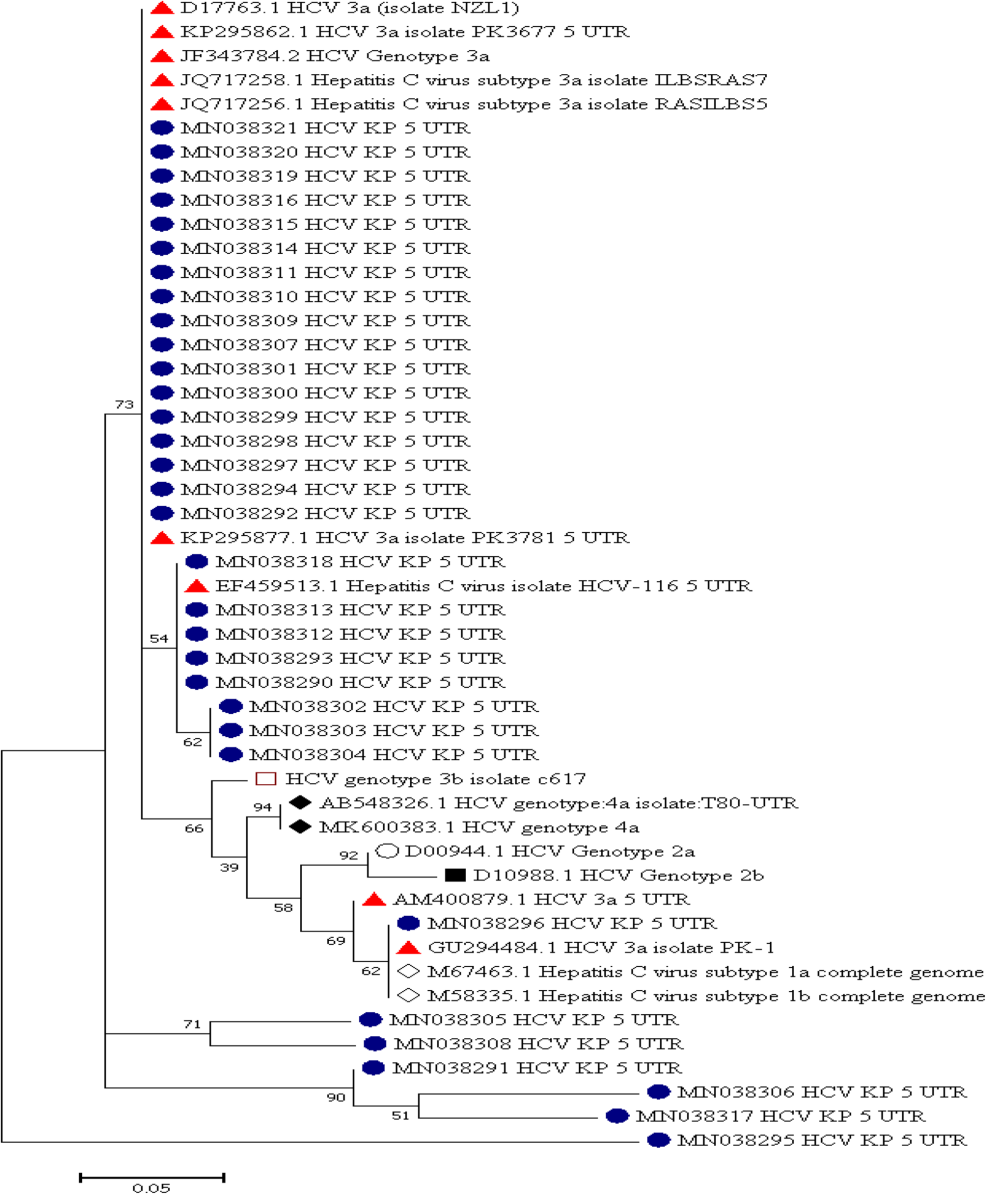
The evolutionary history was inferred via the maximum likelihood method, and the tree with the highest log likelihood (− 421.51) is displayed. The percentage of trees in which the linked taxa clustered together is presented next to the branches. All positions with absent data and gaps were removed. There were a total of 84 positions in the final dataset. Evolutionary analyses were performed by MEGA7 and HCV 5′ UTR sequences with reported genotypes. The highlighted samples show different genotypes, and genotype 3 (highlighted in red) was predominant and matched with the reference genotypes.

Out of all the patients (n = 44), sequencing was successful for samples from 32 patients, and the remaining 12 (8 mild, 2 moderate and 2 gross) patients had sequences that showed less similarity during BLAST because of their small size/nucleotide bp and hence were excluded. The sequence alignment of the 5ʹ-UTR isolates (entitled Hepacivirus C isolate AIMZ7 KP 5ʹ-UTR) was performed with reference genotype (1–7) sequences from the database. The well-conserved areas and few nucleotide substitutions in the 5ʹ-UTR of the HCV genome are shown in Fig. 2. The data also revealed that the length of the 5ʹ-UTR of HCV was up to 183 nucleotides. In the phylogenetic tree, most of the isolates were in a clad and clustered perfectly with the reference sequence. The two isolates from the group did not cluster with the reference sequence. The phylogenetic tree indicated that most of the isolates clustered with subtypes 3a and 3b, and few of them clustered with other reference genotype sequences (Fig. 3).

**Figure 4:**
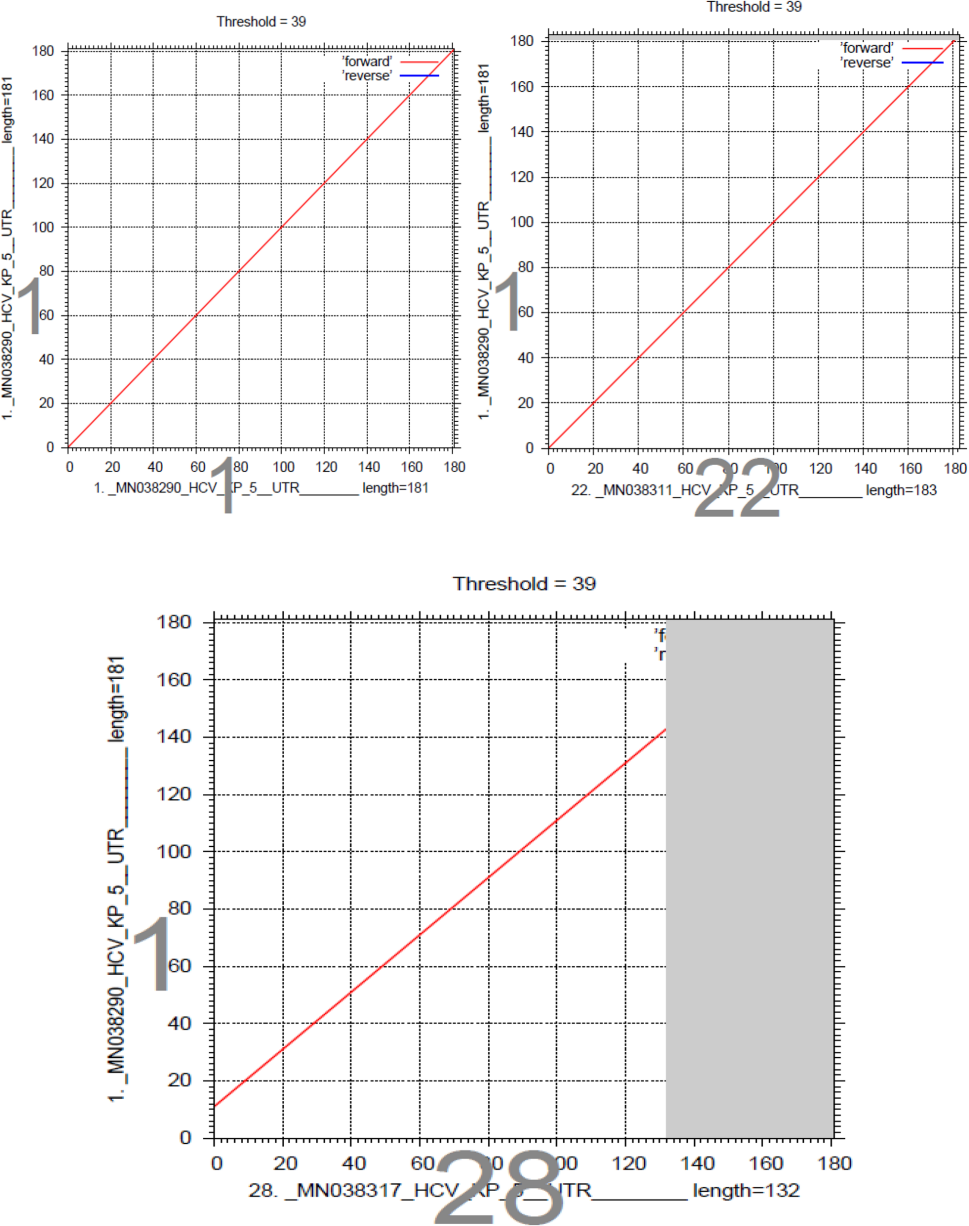
Sequencing of the HCV 5ʹ UTR was performed with a forward primer (red color), and the nucleotide similarity of the 5ʹ UTR sequence was checked by using multiple alignment fast Fourier transform (MAFFT version 7 software: https://mafft.cbrc.jp/alignment/server/large.html). All the sequences show significant similarity with sample 1, and sequence no. 22 shows significant similarity with sequences no. 1 and 28.

Sequencing of the HCV 5ʹ-UTR was performed with a forward primer (red color), and the nucleotide similarity of the 5ʹ-UTR sequence was checked by using multiple alignment fast Fourier transform methods (MAFFT version 7 software: https://mafft.cbrc.jp/alignment/server/large.html). All the sequences show significant similarity with sample and sequence no. 22, and sequence 28 shows significant similarity with sequence no. 1, as shown in Fig. 4.

## Discussion

**Figure 5:**
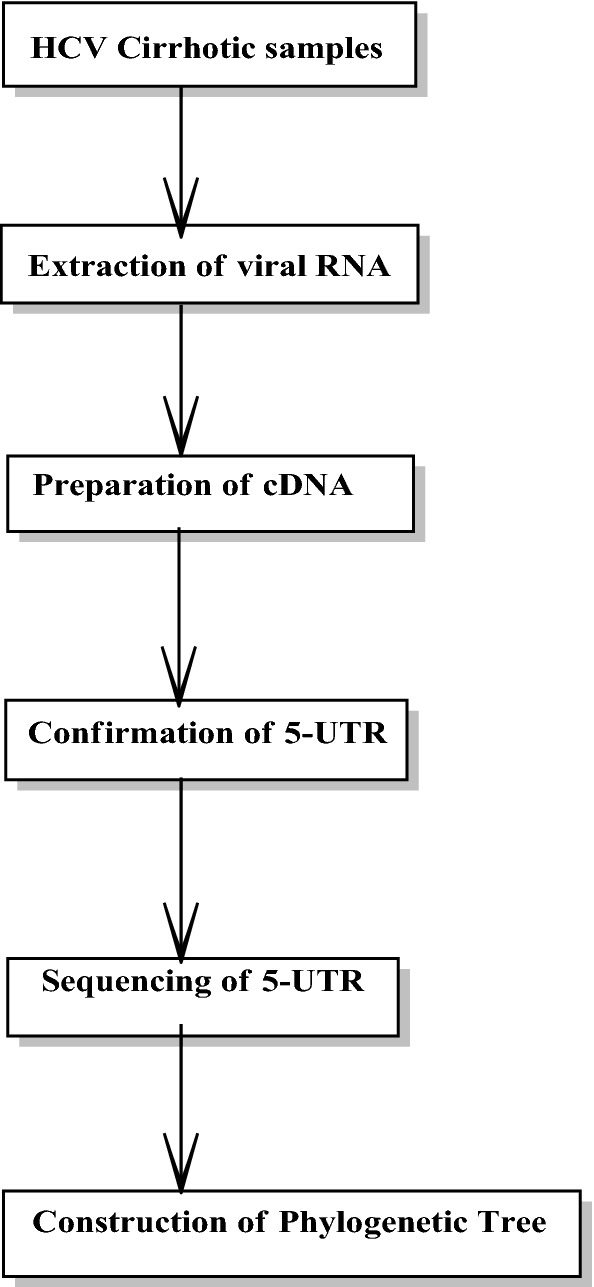
Flow chart of the phylogenetic tree analysis of HCV cirrhotic patients.

Hepatitis C virus (HCV) has a broad range of genotypes and quasispecies due to frequent genetic mutations in its RNA. The identification of HCV genotypes through direct sequencing of the 5ʹ-UTR (Fig. 5) is a great technique because it does not require pattern processing steps and uses amplified products obtained from one-step, nonnested PCR. Additionally, direct sequencing of PCR products provides more comprehensive sequence information than different genotyping analyses. Different studies have reported that the HCV 5ʹ-UTR of 324 to 341 nucleotide sequences is the most conserved region^22^ and can be used for the sequence-based identification of HCV genotypes^17–23^.

The purpose of the current research work was to identify different HCV genotypes from the 5ʹ UTR isolated from patients of the Khyber Pakhtunkhwa (KP) region of Pakistan. The results showed that different genotypes were present in the phylogenetic tree, and genotype 3 was noted as the most predominant. Genotype 3 (a, b) was reported in 26 (59%) isolates of the phylogenetic tree and coincided with the position of each genotype 1, 2, and 4. Sequence conservation in the 5ʹ-UTR HCV genome is vital for the detection of genotypes by sequence evaluation from samples that are not typed with more generally used assays. In the geographic area of Pakistan, HCV genotypes have varied distributions. Genotype 3 was detected more than the other genotypes in this area, as clearly stated in the study of Idress and Riazudin^24^.

Another finding of the study was that genotypes 1 and 2 are also found in this area. Genotypes 1 and 2 are mostly found in Europe and East Asian countries. The detection of these genotypes in KP revealed the immigration of people from one state to another^25^. The rise of genotypes 1 and 2 also causes major problems during treatment, as these patients show less response to antiviral drugs^26^. The occurrence of genotype 2 was also previously reported but was very rare in the local province and neighboring states of Pakistan^24^.

One of the main important findings of our results was that genotype 4 was found very rarely in our state. Recently, HCV genotype 4 has spread in parts of Europe due to variations in the populace structure, ways of transmission and migration. The characteristics of genotype 4 infection and proper therapeutic programs are not well described^27^. Genotype 4 is mostly found in Northern Africa and the Middle East (Egypt, Iran). As these are neighboring countries of Pakistan, immigration could be a factor^28–30^. In general, in this study, the number of samples was small, and available funding was limited. Further study with a larger number of samples is necessary to completely identify the genotypes of this region.

## Conclusion

The study concludes that genotype 3a is the predominant genotype of HCV in the Khyber Pakhtunkhwa region, Pakistan. Direct sequencing of the 5ʹ UTR is a valuable method for clinical detection of different HCV genotypes. Genotype 4 was rarely detected in the tribal area of Khyber Pakhtunkhwa. I am very thankful to the gastroenterology laboratories of tertiary hospitals of Peshawar and the diagnostic laboratory of the Centre of Biotechnology and Microbiology University of Peshawar, Pakistan. I am highly thankful to the Institute of Pathology, in particular to the Laboratory of Translational Molecular Pathology (University Hospital of Cologne Germany) and Prof. Dr. Margarete Odenthal for the helpful discussions and in proofreading of the manuscript.
